# Evaluation of a wrist-worn photoplethysmography monitor for heart rate variability estimation in patients recovering from laparoscopic colon resection

**DOI:** 10.1007/s10877-022-00854-w

**Published:** 2022-04-08

**Authors:** Juha K. A. Rinne, Seyedsadra Miri, Niku Oksala, Antti Vehkaoja, Jyrki Kössi

**Affiliations:** 1 Department of Surgery, Päijät-Häme Central Hospital, Tampere University, Lahti, Finland; 2 Finnish Cardiovascular Research Center - Tampere, Tampere University, Arvo Ylpön Katu 34 (33520 Tampere), P.O. Box 100, FI-33014 Tampere, Finland; 3 Faculty of Medicine and Health Technology, Tampere University, Arvo Ylpön Katu 34 (33520 Tampere), P.O. Box 100, FI-33014 Tampere, Finland; 4 Vascular Centre, Tampere University Hospital, Elämänaukio 2 (33520 Tampere), P.O. Box 2000, 33521 Tampere, Finland; 5 PulseOn Ltd, Tekniikantie 12, 02150 Espoo, Finland

**Keywords:** Photoplethysmography, PPG, Holter monitor, Heart rate variability, Postoperative recovery, Inter-beat-intervals, RR intervals

## Abstract

To evaluate the accuracy of heart rate variability (HRV) parameters obtained with a wrist-worn photoplethysmography (PPG) monitor in patients recovering from minimally invasive colon resection to investigate whether PPG has potential in postoperative patient monitoring. 31 patients were monitored for three days or until discharge or reoperation using a wrist-worn PPG monitor (PulseOn, Finland) with a Holter monitor (Faros 360, Bittium Biosignals, Finland) as a reference measurement device. Beat-to-beat intervals (BBI) and HRV information collected by PPG were compared with RR intervals (RRI) and HRV obtained from the ECG reference after removing artefacts and ectopic beats. The beat-to-beat mean error (ME) and mean absolute error (MAE) of good quality heartbeat intervals obtained by wrist PPG were estimated as − 1.34 ms and 10.4 ms respectively. A significant variation in the accuracy of the HRV parameters was found. In the time domain, SDNN (9.11%), TRI (11.4%) and TINN (11.1%) were estimated with low relative MAE, while RMSSD (34.3%), pNN50 (139%) and NN50 (188%) had higher errors. The logarithmic parameters in the frequency domain (VLF Log, LF Log and HF Log) exhibited the lowest relative error, and for non-linear parameters, SD2 (7.5%), DFA α1 (8.25%) and DFA α2 (4.71%) were calculated much more accurately than SD1 (34.3%). The wrist PPG shows some potential for use in a clinical setting. The accuracy of several HRV parameters analyzed post hoc was found sufficient to be used in further studies concerning postoperative recovery of patients undergoing laparoscopic colon resection, although there were large errors in many common HRV parameters such as RMSSD, pNN50 and NN50, rendering them unusable.

ClinicalTrials.gov Identifier: NCT04996511, August 9, 2021, retrospectively registered

## Introduction

Heart rate variability (HRV) is widely used to assess the recovery of athletes after physical training [1][2]. It has also been applied in clinical settings to monitor the recovery of patients and detection of complications, e.g. in cardiac surgery [3], sepsis [4, 5] and anesthesia and intensive care [6]. A few studies have focused on the use of HRV monitoring in gastrointestinal surgery [7–10]. These studies have suggested that HRV reflects surgical trauma [7], is correlated with the amount of intraoperative blood loss and number of postoperative complications [8] and that HRV parameters show a transient decrease after surgery with a return to baseline by the third postoperative day in patients without complications [9].

The accuracy of beat-to-beat heart rate information collected by photoplethysmography (PPG) has been previously evaluated in several studies and in different patient groups [11–14]. In a study by Tarniceriu et al. [11] patients were monitored postoperatively, and the beat-to-beat intervals (BBI) measured by PPG were compared with an ECG reference. The beat-to-beat mean absolute error for the sinus rhythm (SR) patient group was 7.34 ms, showing a reasonable correlation with the ECG reference. In another study by Parak et al. [15], HRV information on 10 healthy subjects was recorded by PPG and a reference ECG device. The beat-to-beat mean absolute error after applying an artifact correction algorithm was 5.94 ms, indicating that PPG provided similar accuracy to ECG. The relative error of PPG for specific HRV parameters has been studied in healthy adults and vascular surgery patients. The most reliable parameters were SDNN and SD2 of a Poincaré plot, showing a relative error of 2.46% and 2%, respectively, while RMSSD was less accurate, showing a relative error as high as 29.89% [14, 16].

One weakness in many of the studies concerning different HRV parameters is that the study subjects were monitored for only a short time, e.g., a few seconds to half an hour, at a certain time of day or in a laboratory setting while resting, or during physical exertion. The short recording time gives only a snapshot of the subject's physiological state and overlooks the normal circadian rhythm with the peak recovery of HRV occurring at the end of the resting period. Continuous monitoring of the patient gives more data points and can therefore provide a more detailed perspective on recovery. However, for continuous monitoring, the measurement technique should be convenient and unobtrusive. Wearable PPG monitors could potentially be one answer.

To the best of our knowledge there are no previous studies using PPG for HRV estimation in abdominal surgery patients. The aim of our study was to evaluate the beat-to-beat interval and HRV estimation accuracy of a wrist-worn PPG monitor (PulseOn, Finland) by comparing the values obtained with those of a Holter monitor in patients recovering from laparoscopic colon resection within an enhanced recovery (ERAS) protocol. We were also interested in the feasibility of conducting measurements in clinical settings. Our hypothesis was that a wrist-wearable PPG monitor can provide HRV indexes with adequate accuracy for monitoring the recovery of patients after abdominal surgery.

## Study subjects

The study subjects were recruited from patients scheduled for laparoscopic colon resection for benign or malignant disease at Päijät-Häme Central Hospital. The study was approved by the ethics committee of Helsinki University Hospital and the Finnish National Supervisory Authority for Welfare and Health (Valvira). Included were patients presenting with any pathology requiring a colon resection and consenting to participate in the study. The study was registered in ClinicalTrial.gov database, identifier: NCT04996511.

Patients with atrial fibrillation or other chronic arrhythmia that would prevent assessment of recovery by monitoring of the autonomic nervous system using HRV analysis were excluded. Patients considered mentally unfit to give informed consent or deemed likely to pose cooperation problems (e.g., dementia, alcoholism, substance abuse) were also excluded. All patients were Caucasian light to fair skinned ethnic Finnish people. There were 17 females and 14 males, median age 71 (28–86) years and median BMI 27 (20–35) kg/m2. Median ASA physical status classification was 2 (1–3) and median Charlson Comorbidity Index (CCI) was 5 (0–9). There were five previous or current smokers and 26 nonsmokers.

All operations were carried out laparoscopically using the 4 trocar technique with specimen extraction via 5–10 cm oblique incision from either the left or the right side of the abdomen. Eighteen operations were for malignant disease and thirteen for benign. Median operation time was 145 min (69–240 min), and median blood loss was 20 ml (10 ml–150 ml). Long-lasting local anesthetic was administered at the incision sites at the end of the operation. Epidural anesthesia was not used. Postoperative treatment was provided according to enhanced recovery after surgery (ERAS) principles including early oral uptake, urinary catheter removal and active mobilization of the patient.

## Methods

ECG electrodes were placed in standardized five-lead configurations either by the first author (JKAR) or a research nurse on the first postoperative morning. A PPG monitor was placed on the wrist with the wristband tightened snugly but not excessively tight to prevent discomfort. Monitoring was continued for two days (until the third postoperative day) or until discharge if earlier, or the time of a complication requiring intervention.

A PulseOn Aino photoplethysmography device (version 1.2, firmware version 1.3.0) was used. It uses two green color LEDs with a peak wavelength of 573 nm and 25 Hz sampling rate.

eMotion Faros 360 (Bittium Biosignals, Finland) firmware version 3.5.1 is a five-lead Holter monitor. Most patients were recorded using 1 kHz sampling frequency, but in ten patients 250 Hz was used due to a configuration error. Ambu BlueSensor L-00- S disposable Ag/AgCl electrodes were used in the recordings. Mason Likar electrode placement was used with the limb lead electrodes; the arm electrodes were located at the shoulders below the clavicle bone and the left leg electrode at the left flank at the height of the 10th rib. The chest electrode was located at the height of the 6th–7th rib five centimeters to the right of the xiphoid process.

For reliable calculation of the reference HRV parameters, the ECG signal was first filtered to remove the baseline wandering of the raw ECG signal as well as the high frequency noise caused by powerline interference. This was done using infinite impulse response (IIR) and median high-pass and low-pass filters, respectively, as well as forward–backward filtering. The RR intervals were then extracted using the MATLAB inbuilt function ‘findpeaks’. The BBI information from the wrist-worn monitor was already extracted by the internal algorithms of the device and PPG inter- beat intervals (IBIs) were initially stored on the memory of the device and later transferred to a mobile device by Bluetooth. Each IBI is accompanied with a signal quality index classifying the IBIs as ‘reliable’ or ‘unreliable’.

The PPG IBIs and ECG RRIs were further processed using an artifact correction algorithm [17]. Ectopic beats were also removed using the algorithm proposed by Thuraisingham et al. [18]. The PPG monitor and the reference Holter devices had different internal clocks resulting in a slight time drift during long-term recordings. The BBIs were therefore synchronized, and the time drift was compensated to enable beat-to-beat error estimation. Each beat of the PPG IBIs marked as “reliable” by the PulseOn algorithm was paired with the corresponding ECG RRIs.

The BBIs and corresponding RRIs were subsequently divided into 5 min windows for calculation of HRV parameters. The windows were shifted in 60 s steps and a set of HRV parameters was calculated for those window pairs where the sum of the reliable BBIs was at least 80% of the length of the window. This was done to ignore any windows which did not contain enough beats for further evaluations. Several HRV parameters in both time and frequency domains as well as non-linear parameters were then calculated for each accepted window pair. The estimated HRV parameters are shown in Table 1 together with brief explanations. MATLAB was used in the parameter calculation.

**Table 1:** Descriptions of the evaluated HRV parameters

SDNN	Standard deviation of NN intervals
RMSSD	Root mean square of successive differences
pNN50	Percentage of intervals having more than 50 ms difference
NN50	Number of intervals having more than 50 ms difference
IQR	Middle spread of intervals i.e., 25 to 75 percentile range
Median	Median of intervals
Mean RR	Mean of RR intervals
Kurtosis	Kurtosis i.e., fourth moment or tailedness of the distribution of the intervals
Variance	Variance of intervals
Mode	Mode of intervals
HR Mean	Mean of heart rate
TRI	Triangular index
TINN	Triangular interpolation of NN intervals
VLF Abs	VLF absolute power
LF Abs	LF absolute power
HF Abs	HF absolute power
LF Norm	LF normalized power*
HF Norm	HF normalized power
VLF Log	Natural logarithm of VLF absolute power
LF Log	Natural logarithm of LF absolute power
HF Log	Natural logarithm of HF absolute power
VLF Rel	VLF relative power
LF Rel	LF relative power
HF Rel	HF relative power
LF/HF	LF power to HF power ratio
ApEn	The approximate entropy
SD1	In Poincaré plot, standard deviation perpendicular to the line-of-identity
SD2	In Poincaré plot, standard deviation along the line-of-identity
SD1/SD2	Ratio of SD1 to SD2
DFA α1	Scaling exponent α1 of detrended fluctuation analysis
DFA α2	Scaling exponent α2 of detrended fluctuation analysis

*LF and HF absolute power were normalized by the difference of VLF and total power (TP) absolute values. The relative power values of LF, HF and VLF were obtained by dividing the VLF, LF and HF absolute power values only by TP

To understand the performance of PPG for HRV parameter monitoring, the whole band and high frequency components of HRV in the time domain such as SDNN and RMSSD were computed. The same was done in the frequency domain by estimating both low-frequency components (LF Log, LF Rel, LF Abs) and high-frequency components (HF Log, HF Rel, HF Abs). Other parameters analyzed were the ratio parameters (LF/HF, SD1/SD2) and non-linear parameters (ApEn, SD1, SD2, DFA α1, DFA α2).

### Statistical methods

The error metrics used in the accuracy evaluation are mean error (ME), mean absolute error (MAE), relative mean absolute error (MAPE), root mean square error (RMSE), relative root mean square error (RMSPE), bias, relative bias and 5th and 95th percentiles of bias of individual 5 min windows. Equations (1–5) were used for the calculation of these error metrics. The standard deviation of differences (beat-to-beat and 5 min segments) was calculated using Eq. (6). Limits of agreement of the Bland–Altman plot were determined using Eq. (7), and in the same plot confidence intervals (CIs) were calculated using Eqs. (8–9).

1 $$ ME = \frac{1}{n}\mathop \sum \limits_{i = 1}^{n} \left( {y_{i} - x_{i} } \right), $$

2 $$ MAE = \frac{1}{n}\mathop \sum \limits_{i = 1}^{n} \left| {y_{i} - x_{i} } \right|, $$

3 $$ MAPE = \frac{100}{n}\mathop \sum \limits_{i = 1}^{n} \left| {(y_{i} - x_{i} )/y_{i} } \right|, $$

4 $$ RMSE = \sqrt {\frac{1}{n}\mathop \sum \limits_{i = 1}^{n} \left( {y_{i} - x_{i} } \right)^{2} }, $$

5 $$ RMSPE = 100\sqrt {\frac{1}{n}\mathop \sum \limits_{i = 1}^{n} \left( {(y_{i} - x_{i} )/y_{i} } \right)^{2} }, $$

6 $$ \sigma = \sqrt {\frac{1}{n}\mathop \sum \limits_{i = 1}^{n} (x_{i} - \mu )^{2} }, $$

7 $$ LoR = bias \pm 1.96*Std, $$

8 $$ 95\%\; CI \;of \;bias = bias \pm t_{{\left( {n - 1,1 - \frac{\alpha }{2}} \right)}} *\left( {\frac{{S_{d} }}{\sqrt n }} \right), $$

9 $$ 95\% \;CI \;of\; LoA = LoA \pm t_{{\left( {n - 1,1 - \frac{\alpha }{2}} \right)}} \sqrt {\left( {\frac{1}{n} + \frac{{1.96^{2} }}{{2\left( {n - 1} \right)}}} \right)S_{d} }, $$

A box plot was used to illustrate the distribution of the subject-wise relative mean absolute error of the HRV parameters as well as the distribution of the coverage of accepted 5 min windows based on the 80% threshold for the cumulative sum of good quality IBIs. The red line in the middle of the box indicates the median value. The thick part of the box indicates the range of the 25th and 75th percentiles. A datapoint is considered an outlier if it is more than 1.5 times the interquartile (25th–75th percentile) range apart from the 25th or 75th percentile limit. The whiskers extent to the most extreme data points are not considered outliers.

## Results

### Beat-to-beat error estimation result

Table 2 presents the results of beat-to-beat error estimation for all subjects. The MAE of beat-to-beat estimates for all subjects was 10.39 ms. The IBI coverage (the percentage of the cumulative sum of good quality IBIs and the recording length) of the PPG was 49.63%.

**Table 2:** The result of beat-to-beat error estimation for all subjects

ME [ms]	MAE [ms]	MAPE [%]	RMSE [ms]	RMSPE [%]	IBI Coverage [%]
− 1.35	10.4	1.29	20.3	2.52	49.6

‘Good quality IBI’ refers to IBIs marked as ‘reliable’ by a proprietary signal quality estimation algorithm of the wrist-worn PPG device

*ME* mean error for all good quality IBIs, *MAE* mean absolute error for all good quality IBIs, *MAPE* relative mean absolute error for all good quality IBIs, *RMSE* root-mean-square error for all good quality IBIs, *RMSPE* relative root-mean-square error for all good quality IBIs, *IBI Coverage* percentage of good quality IBIs from all IBIs

### HRV parameter error estimation result

The error parameters in Table 3 show that the accuracy of HRV parameters varies significantly. In the time domain, the relative MAE for the basic parameter accounting for the overall HRV power SDNN is 9.11%. However, the MAE for RMSSD, which emphasizes the high-frequency components of HRV, is 34.28%. The estimate of the relative mean absolute error of the statistical time domain parameters such as pNN50 and NN50 had a very high error, largely due to the low values of pNN50/NN50 of the reference value in this study subject group (close to 0). In the frequency domain, the relative MAE of parameters in the logarithmic domain (3.6% for LF and 6.5% for HF) was much lower than the values for absolute LF and HF power (23.1% and 54.3%, respectively). For non-linear parameters, except for SD1 (34.29%), the relative MAE exhibited was relatively low (7.54% for SD2, 8.25% for DFA α1, 4.71% for DFA α2 and 8.21% for ApEn). On the other hand, the estimates for ratio parameters such as SD1/SD2 and LF/HF resulted in high relative MAEs of 37.2% and 28.7%, respectively.

**Table 3:** The error parameters of HRV parameters in different domains. The error parameters were obtained by taking the average of accepted windows, i.e., windows fulfilling the criteria of at least 80% (4 min) cumulative sum of good quality IBIs

Parameters		MAE	MAE (%)	RMSE	RMSE (%)	P05	P95	Bias	Bias (%)	SD
SDNN	[ms]	2.77	9.12	5.26	14.6	− 14.5	2.38	− 0.67	1.93	5.42
RMSSD	[ms]	4.29	34.2	5.34	43.2	− 1.16	6.07	3.61	32.3	2.21
pNN50	[%]	1.98	139	2.81	264	− 1.41	4.22	0.91	94.9	1.91
NN50	[beats]	5.79	188	8.47	332	− 5.00	14.8	2.27	153	6.26
IQR	[ms]	3.58	9.58	6.48	14.0	− 19.4	4.35	− 0.58	2.32	6.84
Median	[ms]	4.08	0.45	4.77	0.53	− 4.00	7.80	0.30	0.035	3.30
Mean RR	[ms]	4.68	0.52	5.97	0.66	− 3.53	13.4	0.95	0.11	5.37
Kurtosis	[–]	0.35	10.1	0.60	16.9	− 0.47	0.34	0.038	2.84	0.37
Variance	[ms]	238	19.8	616	45.1	− 1450	142	− 126	6.95	498
Mode	[ms]	13.2	1.47	19.4	2.20	− 40.3	34.7	− 0.91	− 0.077	23.1
HR Mean	[BPM]	0.35	0.51	0.45	0.65	− 1.18	0.32	− 0.07	− 0.10	0.48
TRI	[–]	0.83	11.4	1.08	14.7	− 1.76	1.73	0.055	3.41	1.26
TINN	[ms]	0.012	11.1	0.017	16.0	− 0.031	0.023	0.002	4.25	0.016
VLF Abs	[ms^2^]	422	20.3	632	28.4	− 1530	85.5	− 376	− 17.4	560
LF Abs	[ms^2^]	140	23.1	203	37.1	− 467	254	− 9.61	3.76	263
HF Abs	[ms^2^]	165	54.3	223	79.7	− 265	189	66.8	43.4	133
LF Norm	[n.u.]	7.24	13.71	9.29	18.45	− 9.65	5.41	− 4.54	− 4.77	4.06
HF Norm	[n.u]	7.24	31.39	9.29	42.66	− 5.41	9.65	4.54	26.21	4.06
VLF Log	[log]	0.27	3.61	0.41	5.38	− 0.80	0.050	− 0.25	− 3.24	0.325
LF Log	[log]	0.23	3.62	0.33	5.25	− 0.60	0.31	− 0.019	− 0.15	0.325
HF Log	[log]	0.35	6.53	0.44	8.59	− 0.50	0.47	0.21	4.47	0.307
VLF Rel	[%]	7.06	12.0	9.73	16.0	− 20.3	− 0.50	− 6.19	− 9.85	6.58
LF Rel	[%]	3.52	23.0	5.19	43.6	− 0.75	14.7	1.91	16.5	4.98
HF Rel	[%]	5.38	71.3	7.16	111	− 2.16	6.63	4.28	67.6	2.63
LF/HF	[n.u.]	0.89	28.7	1.23	36.6	− 1.19	0.25	− 0.74	− 11.8	0.421
ApEn	[–]	0.077	8.22	0.10	12.5	− 0.093	0.080	0.027	3.80	0.052
SD1	[ms]	3.03	34.3	3.78	43.2	− 0.824	4.29	2.55	32.4	1.56
SD2	[ms]	3.49	7.55	7.28	13.2	− 21.7	2.43	− 2.00	− 1.63	7.73
SD1/SD2	[–]	0.10	37.2	0.13	49.4	− 0.021	0.13	0.097	35.8	0.046
DFA α1	[–]	0.14	8.26	0.17	10.3	− 0.046	0.19	0.11	6.83	0.072
DFA α2	[–]	0.089	4.72	0.13	7.43	− 0.063	0.16	0.058	3.19	0.097

In total, 21,366 windows (approximately 35% of the total number of windows) were accepted after fulfilling the primary condition. The estimated measurements through PPG and ECG were compared using Eqs. (2–5). 5th and 95th percentiles are the cut-off points where 5% and 95% of mean differences (Eq. (1)) lie below them, respectively. Bias was obtained using Eq. (1) and its percentage was calculated by dividing the mean error of paired segments by the values obtained from the reference device. SD (Eq. (8)) is the standard deviation of the bias

**Fig. 1:**
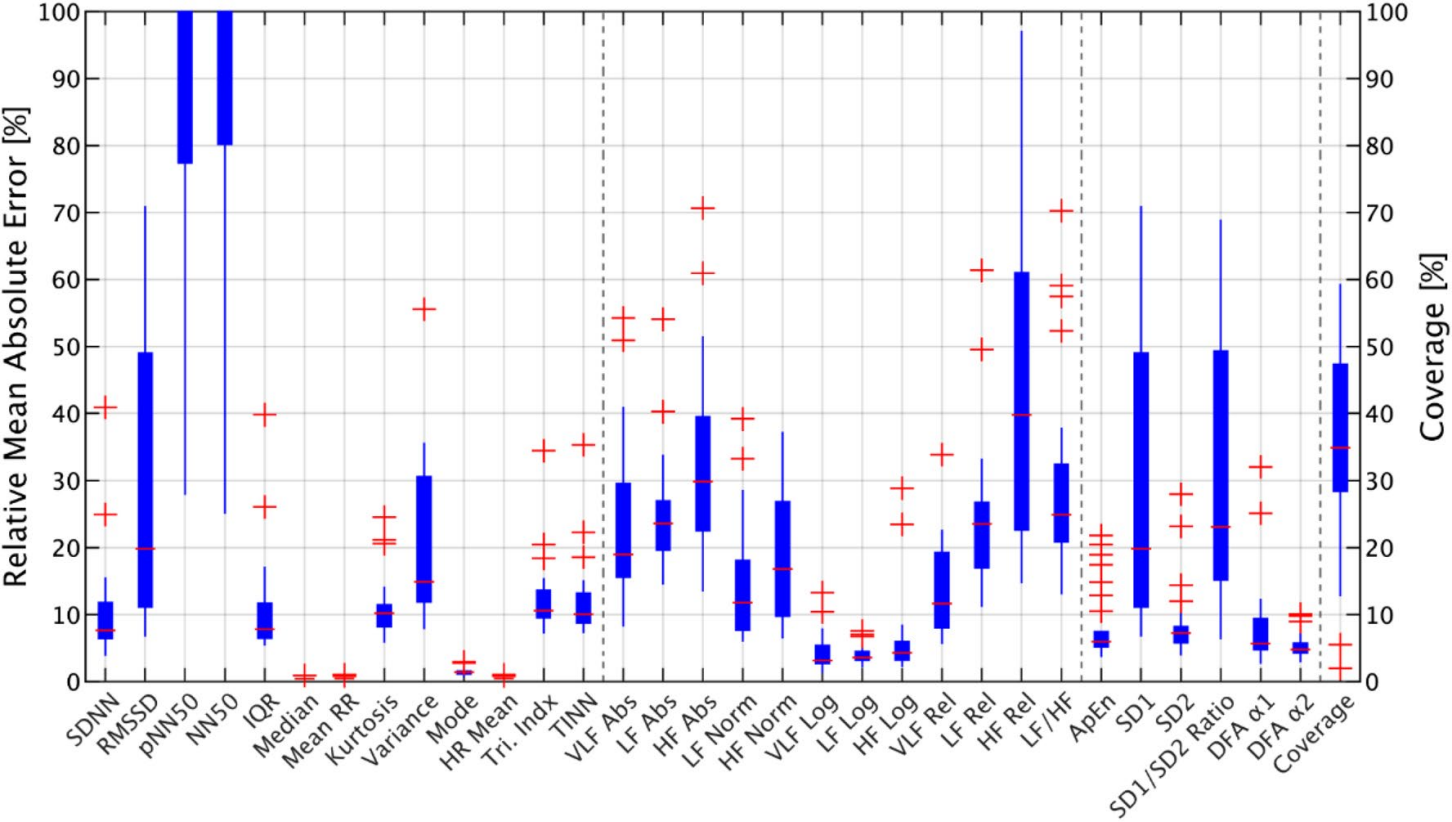
The distribution of the average relative MAE for all subjects. Each datapoint in the bar chart represents one subject. Time domain, frequency domain and non-linear parameters are separated by dashed lines. The bottom and upper part of boxplots, the red dashes and crosses represent 25th and 75th percentiles, median values and outliers respectively. The right bar represents the percentage of accepted windows for each subject, the median which is around 35%, which means that the condition that was set at the beginning led to the acceptance of around 35% of windows on average

Figure 1 presents the subject-wise distribution of MAPE of the HRV parameters evaluated and the percentage of accepted windows after applying the 80% minimum threshold for a cumulative good quality IBI sum. With this threshold the median coverage of accepted 5 min windows was 35%, the 25% and 75% inter-quartile ranges being between 28 and 47%. For two subjects the coverage of accepted windows was less than 10%, the highest coverage being at 60%. The figure does not show the relative MAE for values more than 100%. The results show that the accuracy of PPG for some individuals is higher than for others and that the 25th percentile values for most parameters are around 10%. On the other hand, estimates of pNN50/NN50 and other parameters associated with the high-frequency components of HRV resulted in a high relative MAE. The coverage of the 5 min windows for which the HRV parameters were reported varied significantly, having a median of 35% and an interquartile range from 28 to 47%. Two patients had an HRV coverage of less than 10%, which indicated that the technique used is not necessarily suitable for everyone. The most likely reason for poor coverage is human error, e.g., the wrist device being worn too loose or an incorrect position of the device on the wrist.

**Fig. 2:**
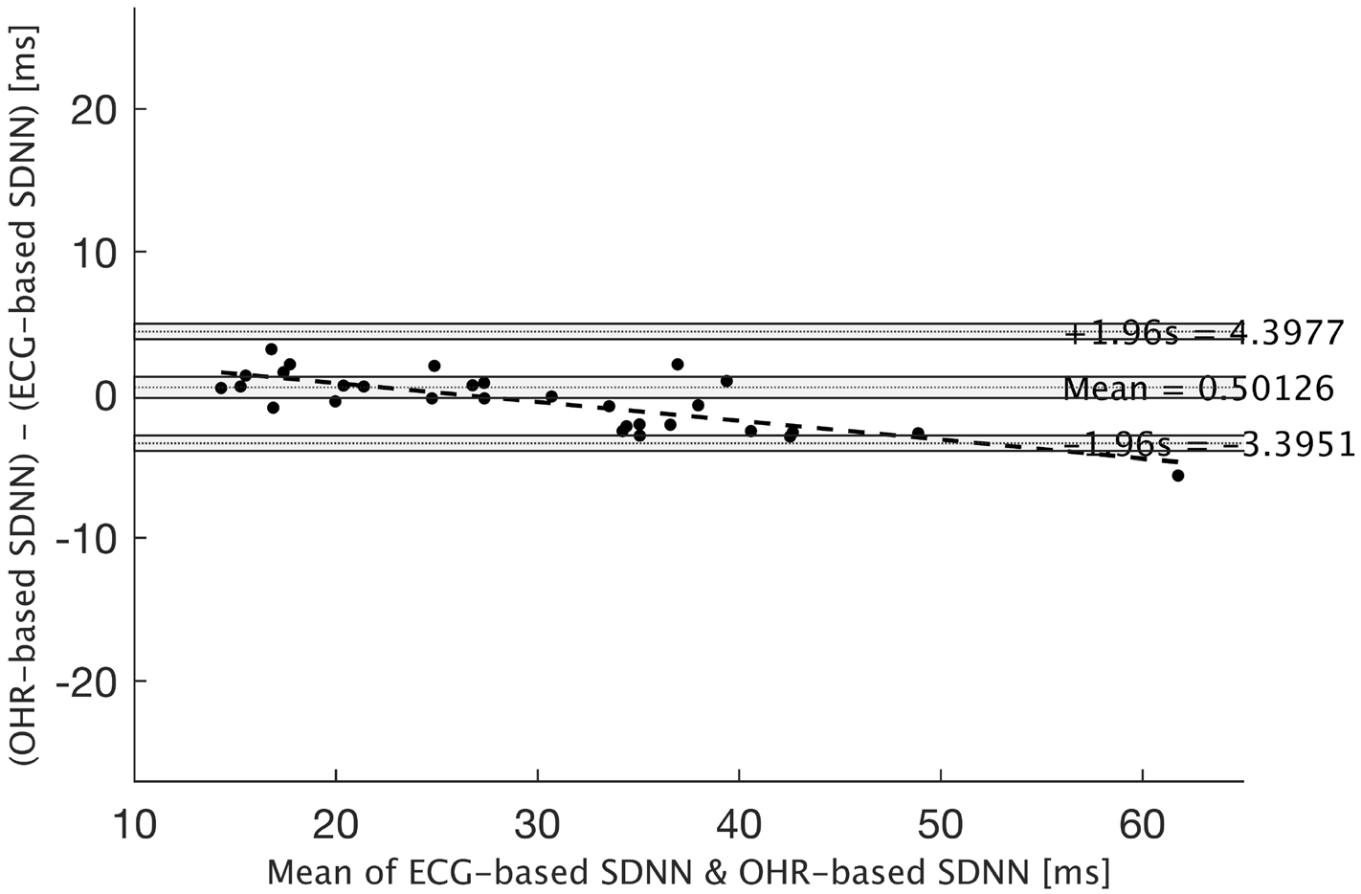
Bland–Altman plot for the SDNN parameter. The mean of bias and limits of agreements (LoA) together with their confidence intervals (CI) are presented. The dashed line with a negative slope is the trend in the datapoints

The Bland–Altman plot of SDNN is depicted in Fig. 2. The negative trend of datapoints indicates the underestimation of SDNN by PPG for higher SDNN values. The zero line is placed within the CIs of the bias, which represents a high agreement between the two measurement methods for SDNN parameters.

**Fig. 3:**
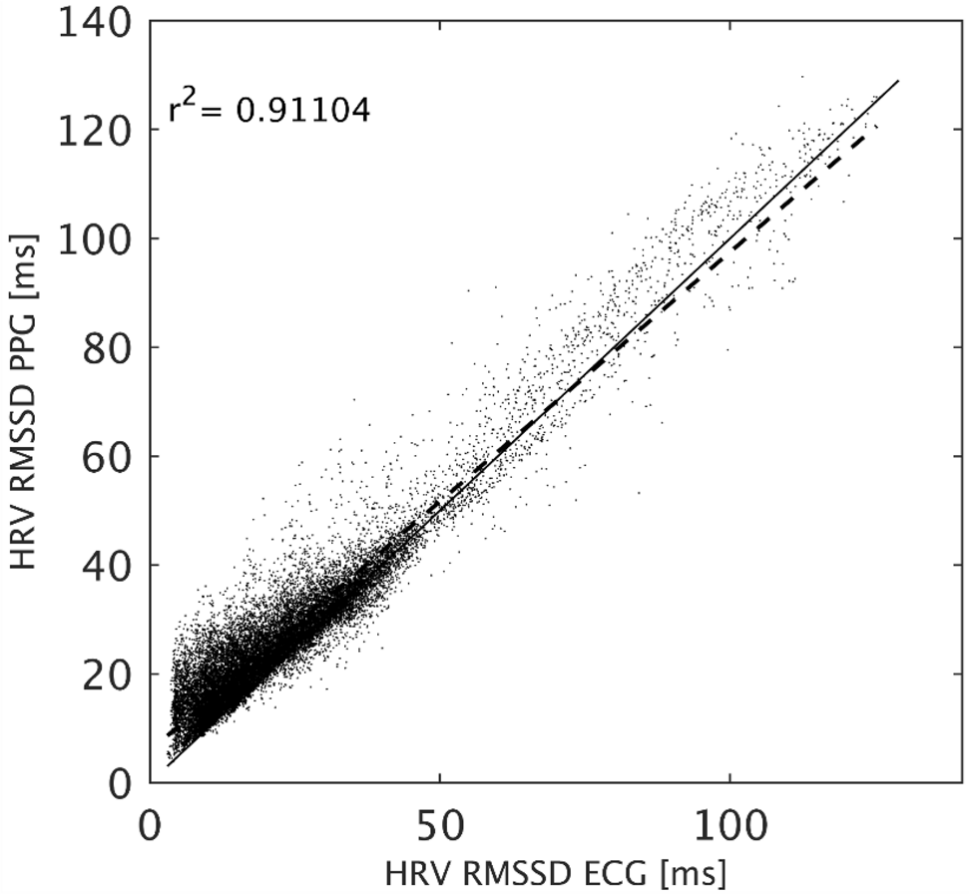
Scatter plot for the RMSSD parameter

Figure 3 presents the scatter plot of RMSSD for all windows of all subjects. Altogether there are 21,315 datapoints having r-squared (r^2^) equal to 0.911. The figure shows that there is a high correlation of accepted windows for both high and low HRV values.

## Discussion

The goal of the present study was to assess the feasibility and accuracy of a wrist-worn PPG in the monitoring of gastrointestinal surgery patients. The main finding is that PPG could potentially be used in postoperative patient monitoring but based on our results this needs to be studied further since there was a high error in some of the parameters measured, rendering them unusable in postoperative patient monitoring. Both the technology and its use in a clinical setting must be validated in larger studies.

In a study by Ushiyama et al. [8], the SDNN value for patients with postoperative complications was 48.7 ± 24.4 ms (average ± standard deviation) while for the “uncomplicated” groups it was 71.2 ± 19.6 ms. Based on the results of this study, these two groups could be accurately distinguished using PPG as MAE and RMSE for SDNN were 2.76 ms and 5.21 ms, respectively. Lerma et al. [19] compared the non-linear HRV parameters of chronic renal failure (CRF) patients measured before hemodialysis with a control group of healthy subjects with comparable demographics. The non-linear HRV parameters differed significantly between the two groups, the SD2 value for the control group being 80.3 ± 19.0 ms, while the SD_2_ for the CRF group was 32.9 ± 14.7 ms. MAE and RMSE results in this study for SD2 were 3.48 ms and 7.27 ms, respectively, indicating that the PPG device used in this study would be able to differentiate between these two groups. Overall, HRV parameters such as SDNN, Tri, TINN, approximate entropy, SD2, DFA α1, DFA α2 and the logarithmic frequency domain HRV parameters exhibited the lowest relative MAE and could potentially be utilized in clinical situations. However, it is beyond the scope of this pilot study to draw any firm conclusions on this.

On the other hand, the ratio parameters as well as certain time (NN50/pNN50) and frequency domain parameters (absolute values) had the highest relative MAE. In a study by Scheffer et al. [10] the patient group with surgical complications had lower pNN50 values (5.71 ± 2.81%), while the uncomplicated group had higher pNN50 values (8.54 ± 5.94%). Based on the findings of the current study, MAE and RMSE for pNN50 were 1.97 percentage points and 2.81 percentage points, respectively, which means that in our study PPG monitor used would not have provided accurate enough data to differentiate the uncomplicated from the complicated patients using pNN50 as indicator. This finding is influenced by methods determining the length of intervals as well as ectopic beat removal methods, and other methods of signal processing should therefore be further investigated. Motion artifacts can greatly affect the quality of PPG signals and investigating ways to minimize the effects of motion artifacts is crucial. Another confounding factor is that the patients with complications were included in our analysis, although our study was underpowered to perform subgroup analysis. This will be addressed in a forthcoming study with a larger study population.

In our study IBI coverage of the PPG device was reasonably good, 49.63%. It should be noted that this was a preliminary study conducted in a busy surgical ward with no prior experience with wrist-worn PPG or Holter monitors. IBI coverage is highly dependent on the amount of movement of the patient, and on subject characteristics such as blood perfusion and skin color [20]. Further, the tightness of the wristband affects the mechanical coupling of the device with the skin and may affect the coverage. These technical issues were new to the study personnel, and it is very probable that in the future IBI coverage will be even better when personnel at the department get more acquainted with the device. Overall, the performance of PPG for the calculation of HRV parameters is dependent on both “subjects” and “parameters”. For example, the SDNN parameter for the subjects who had lower SDNN values was estimated more accurately by the PPG, while the opposite was true for RMSSD. Also, the occasional large errors in BBIs—even after the signal quality analysis—that are caused by motion artifacts could be the reason why some certain parameters such as pNN50 were estimated less accurately than others (pNN50 is more prone to be miscalculated than, say, SDNN). It is also suggested that the effects of using segments of different lengths (other than 5 min) or setting other conditions for accepting segments be further investigated.

Although the coverage of the reported 5 min HRV segment is on average around one-third, it is sufficient for patient monitoring as there is no need to obtain HRV parameter values all the time. In fact, due to the sensitivity of the PPG monitor used to movement artefacts, there has likely been more movement during the discarded segments and therefore these segments would not have provided representative HRV parameter values in the first place. The segments containing an adequate amount of good quality data are likely recorded when the subject has been stationery and calm, thus better representing the status of the autonomic nervous system.

## Conclusions

We were able to demonstrate that the accuracy of several HRV parameters obtained with an unobtrusive wrist-worn PPG monitor evaluated in 5 min segments shows enough potential for PPG to be studied further. The PulseOn photoplethysmography monitor used in the study performed satisfactorily in clinical settings. The large error in some of the common HRV parameters and the high individual variation in accuracy need to be addressed. Both the technology and the use in a clinical setting must be validated in larger studies.
